# Effects of exercise on circadian rhythms in humans

**DOI:** 10.3389/fphar.2023.1282357

**Published:** 2023-10-11

**Authors:** Bingyi Shen, Changxiao Ma, Guanlin Wu, Haibin Liu, Lihong Chen, Guangrui Yang

**Affiliations:** ^1^ School of Bioengineering, Dalian University of Technology, Dalian, China; ^2^ School of Clinical Medicine, Shanghai University of Medicine & Health Sciences, Shanghai, China; ^3^ School of Kinesiology and Health Promotion, Dalian University of Technology, Dalian, China; ^4^ Health Science Center, East China Normal University, Shanghai, China

**Keywords:** exercise, non-photic zeitgeber, biological clock, circadian rhythm, health

## Abstract

The biological clock system is an intrinsic timekeeping device that integrates internal physiology and external cues. Maintaining a healthy biological clock system is crucial for life. Disruptions to the body’s internal clock can lead to disturbances in the sleep-wake cycle and abnormalities in hormone regulation, blood pressure, heart rate, and other vital processes. Long-term disturbances have been linked to the development of various common major diseases, including cardiovascular diseases, metabolic disorders, tumors, neuropsychiatric conditions, and so on. External factors, such as the diurnal rhythm of light, have a significant impact on the body’s internal clock. Additionally, as an important non-photic zeitgeber, exercise can regulate the body’s internal rhythms to a certain extent, making it possible to become a non-drug intervention for preventing and treating circadian rhythm disorders. This comprehensive review encompasses behavioral, physiological, and molecular perspectives to provide a deeper understanding of how exercise influences circadian rhythms and its association with related diseases.

## 1 Introduction

Biological rhythms refer to repetitive processes that occur over specific periods of time, such as a day, a month, or a year. The circadian rhythm, which has a period of approximately 24 h, is particularly relevant to human health. Numerous behavioral, physiological, and biochemical activities demonstrate obvious circadian rhythms. For instance, the sleep-wake cycle, as well as daily changes in blood pressure, heart rate, and body temperature, are widely recognized to display discernible circadian patterns. When the internal biological clock becomes desynchronized with the external environment, it may lead to a mismatch between central and peripheral clocks, as well as between different tissues or organs, and even between different clock genes within the same organ (Reppert and Weaver, 2002). Long-term circadian disturbances are closely linked to the onset and progression of a variety of mental and physical diseases, including cardiovascular diseases, metabolic syndrome, neurodegenerative diseases, and tumors (Fatima and Rana, 2020; Meyer et al., 2022; Bolshette et al., 2023; Lane et al., 2023).

**FIGURE 1:**
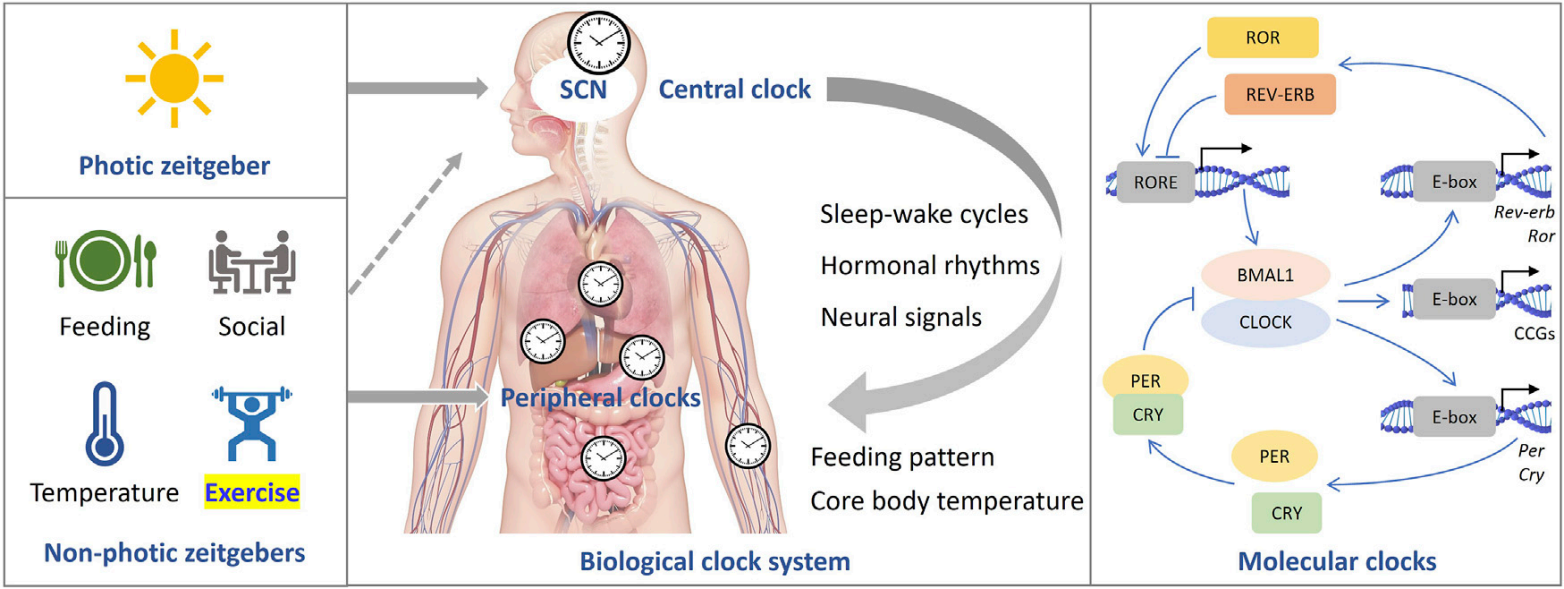
The mammalian clock system. The central clock is situated within the suprachiasmatic nucleus (SCN), where it governs peripheral clocks throughout the body (center). Light serves as the primary zeitgeber, while non-photic cues also have the capacity to synchronize circadian rhythms (left). At the molecular level (right), a set of core clock genes and proteins collaboratively form highly conserved transcriptional-translational feedback loops (TTFL).

In mammals, the central pacemaker of circadian rhythms is located in the suprachiasmatic nucleus (SCN). SCN output is relayed through the nuclei of the hypothalamus thereby transmitting circadian signals to the brain regions that regulate the sleep-wake cycles and synchronizing the peripheral circadian rhythms of other tissues through neurohormonal mechanisms. At the molecular level, the core clock genes and proteins form a set of highly conserved transcriptional-translational feedback loops (TTFL) that have both positive and negative regulatory elements (Figure 1) (Chen and Yang, 2014; Fagiani et al., 2022). The transcription factors, BMAL1 and CLOCK form heterodimers, which bind to the E-box located in the promoter regions of the *per* and *cry* genes to promote the production of PER and CRY proteins. When they accumulate to a certain level in the cytosol, PER and CRY translocate to the nucleus to inhibit the activity of the BMAL1/CLOCK complex, thus repressing their own expressions. In addition, a second feedback pathway composed of nuclear receptors ROR and REV-ERB is involved in promoting and inhibiting the expression of BMAL1, respectively. These core clock components regulate hundreds of other genes, called clock-controlled genes (CCGs) in a circadian manner. Generally, core clockwork mechanisms exist in most tissues and cells of the body, but the expression of CCGs varies by cell type (Koike et al., 2012).

While the circadian rhythm continues to function under constant conditions, such as constant darkness (DD), it can be affected by environmental changes and spontaneous activities like light exposure, diet, and exercise. These daily changes are referred to as zeitgebers and can entrain or reset the circadian rhythms. Among them, light is the most potent zeitgeber (Fisk et al., 2018; Ruan et al., 2021). Non-photic factors including medication, temperature, diet, and exercise can also entrain circadian rhythms. For example, consuming food during specific time affects the transcription levels of clock genes (Ulgherait et al., 2021; Deota et al., 2023), which can aid in maintaining normal circadian rhythms, mitigating metabolic disorders caused by a high-fat diet (HFD) (Hatori et al., 2012; Chaix et al., 2014; Li, 2022), improving the body’s ability to cope with circadian disruption (Ren et al., 2021; Ren et al., 2022), enhancing running endurance without prior exercise (Xin et al., 2023), and even prolonging lifespan (Acosta-Rodriguez et al., 2022).

Over the past few years, exercise has garnered increasing attention as a significant non-photic zeitgeber. Physical inactivity is recognized as a risk factor for numerous common illnesses, including cardiovascular diseases, metabolic disorders, neurodegenerative disorders, and tumors (Schloss et al., 2020; Valenzuela et al., 2020). At the molecular level, exercise has been shown to modulate the expression of clock genes (Martin et al., 2023; Sato and Yamanaka, 2023). Both aerobic and resistance exercise upregulate the expression of *BMAL1* and *PER2* in skeletal muscle (Zambon et al., 2003; Wolff and Esser, 2019). Regular exercise is a healthy lifestyle, in part because it helps keep the biological clock running properly. Similar to light exposure, the timing of exercise affects the circadian rhythm (Youngstedt et al., 2019). Thus, exercise is expected to be a non-invasive, non-pharmaceutical intervention to facilitate the regulation of circadian rhythms. However, the best time of day for strength and endurance training to improve health remains unclear (Bruggisser et al., 2023). This review summarizes relevant literature and discusses two aspects: 1) the impact of exercise on circadian rhythms; and 2) the association between exercise and circadian disorders and related illnesses.

## 2 Effects of exercise on circadian rhythms

Establishing a correlation between exercise and circadian rhythms based on human studies is challenging due to the varying intensity, mode, and duration of exercise. Exercise can be classified as either aerobic or resistance training depending on the energy-producing systems and weight-bearing conditions, or continuous exercise and intermittent exercise based on the length of rest periods. In 2020, the *World Health Organization guidelines on physical activity and sedentary behaviour* underscored the pivotal role of regular physical activity in preventing and treating non-communicable diseases, recommending that adults engage in at least 150–300 min of moderate-intensity or 75–150 min of vigorous-intensity aerobic physical activity per week (Bull et al., 2020). Therefore, the majority of studies investigating the relationship between exercise and circadian rhythm have focused on moderate to high-intensity aerobic exercise lasting more than 30 min per day. This chapter reviews the associations of the timing of exercise with circadian rhythms under normal, constant, and disturbed light conditions (Table 1).

**TABLE 1:** Effects of exercise on human circadian rhythm.

Light	Exercise protocol			Result	Note	References
	Type	Mode	Intensity			
Normal (<10 lux during saliva sampling)	Treadmill	5 d; 1 time/d; 30 min Ex	70% VO_2peak_	Morning exercise: DLMO←(0.49 ± 0.25 h,0.54 ± 0.29 h) in both earlier and later chronotypes	Exercise was performed in the morning (10 h after DLMO) or evening (20 h after DLMO)	Thomas et al. (2020)
				Evening exercise: DLMO→(0.41 ± 0.29 h) in earlier chronotypes, DLMO←(0.46 ± 0.25 h) in later chronotypes		
Normal	Resistance training	60 d; 3 times/wk; Lumbar and lower limb muscle group training	70%–80% of maximum muscle strength	C: Temperature←(0.45 ± 0.40 h)	Resistance training was performed at one of four 45-min time slots between 10 a.m. and 1 p.m	Mendt et al. (2021)
Dim, <10 lux	Cycle ergometer	1 d; 1 time/d; 4 cycles (15 min Ex + 15 min Re)	HR: 140/min	CT8: M_peak_→(0.35 ± 0.13 h)		Miyazaki et al. (2021)
				CT17: M_onset_→(0.45 ± 0.15 h); M_peak_→(0.45 ± 0.12 h); M_offset_→(0.62 ± 0.21 h)		
Dim, <10 lux	Cycle ergometer	4 d; 1 time/d; 10 min Wa +45 min Ex + 10 min Re + 45 min Ex + 10 min Co	65%–75% HR_max_	C: M_onset_→(1.1 ± 0.7 h); M_peak_→(0.8 ± 0.4 h)		Yamanaka et al. (2015)
				CT3: M_onset_→(1.2 ± 1.0 h); M_peak_→(1.0 ± 0.3 h)		
				CT10: M_onset_→(1.3 ± 0.9 h); M_peak_→(1.0 ± 0.5 h); M_offset_→(1.0 ± 0.8 h)		
Dim, <100 lux	Arm and leg exerciser	1 d; 1 time/d; LE: 5 cycles (15 min Ex1 + 15 min Ex2 + 6 min Re); HE: 10 min Wa +40 min Ex + 10 min Co	LE: 60% VO_2max_ + 40% VO_2max_	LE: TSH_onset_→(1.30 ± 0.17 h); M_onset_→(1.05 ± 0.13 h)	LE and HE were performed at CT16 and CT17 respectively	Buxton et al. (1997)
			HE: 75% VO_2max_	HE: TSH_onset_→(1.58 ± 0.32 h); M_onset_→(0.92 ± 0.25 h)		
Dim, 42 ± 19 lux	Stairclimber	1 d; 1 time/d; 10 min Wa +40 min Ex + 10min Co	75% VO_2max_	CT1.5: M_onset_→(0.38 ± 0.23 h)		Buxton et al. (2003)
				CT5: M_onset_→(0.72 ± 0.20 h)		
				CT10.5: M_onset_←(0.50 ± 0.25 h)		
				CT16.5: M_onset_→(0.42 ± 0.23 h)		
Dim, <50 lux (Wake period); <0.5lux (Sleep period)	Treadmill	3 d; 1 time/d; 60 min Ex	65%–75% HRR	1:00: aMT6s Acropgase→	Each participant followed an ultrashort sleep-wake cycle (60 min wake/30 min sleep) for up to 5½ days and exercised at one of eight counterbalanced times of day or night	Youngstedt et al. (2019)
				16:00: aMT6s Acropgase←		
				7:00,13:00 and 16:00: aMT6s Onset←		
				19:00 and 22:00: aMT6s Onset→		
Dim, <10 lux	Cycle- and rowing-type ergometers	12 d; 2 times/d, 4 cycles (15 min Ex + 15 min Re)	HR: 140/min	E: M_peak_←(1.60 ± 0.42 h)	A forced sleep-wake schedule with a period of 23 h and 40 min (8-h rest and 15 h 40-min wake periods) was imposed for 12 cycles. Exercise was performed at CT3 and CT7	Miyazaki et al. (2021)
Dim, <10 lux	Cycle ergometer	4 d, 2 times/d, 4 cycles (15 min Ex + 15 min Re)	65%–75% HR_max_	C&E: M_peak_→ after shift schedule; M_peak_→ in C and recovered in E after free-run	The sleep schedule was phase-advanced by 8 h for 4 days, which was followed by a free-run session for 6 days, and exercise was performed at CT3 and CT7	Yamanaka et al. (2010)
Two groups	Cycle ergometer	3 d, 1 time/d, 6 cycles (Ex 15 min/h)	50%–60% HR_max_	Dim light: Temperature minimum →(7.9 ± 1.0 h, 7.7 ± 2.7 h) in C and CT7	The sleep schedule was phase-delayed by 9 h for 8 days	Baehr et al. (1999)
1. Dim, <500 lux				Bright light: Temperature minimum →(4.8 ± 12.9 h, 5.7 ± 3.2 h) in C and CT7		
2.During exercise, 40 min (5,000 lux) + 20 min (<500lux)						
Dim, <5 lux	Cycle ergometer	7 d, 3 times/d, 45 min Ex	65%–75% HR_max_	C: M_onset_→(1.67 ± 0.45 h); M_offset_→(1.51 ± 0.55 h)	The sleep schedule was phase-delayed by 9 h for 7 days. Exercise was performed at CT8.75, CT10.50 and CT12.25	Barger et al. (2004)
				E: M_onset_→(3.17 ± 0.49 h); M_offset_→(3.51 ± 0.55 h)		
Bright, >5,000 lux	Cycle ergometer	4 d, 2times/d, 4 cycles (15 min Ex + 15 min Re)	65%–75% HR_max_	C: Sleep onset←(4.3 ± 3.8 h) after shift schedule; Sleep onset→(3.6 ± 3.4 h) in the free-run session	The sleep schedule was phase-advanced by 8 h for 4 days, which was followed by a free-run session for 6 days, and exercise was performed at CT3 and CT7	Yamanaka et al. (2014)
				E: Sleep onset←(6.1 ± 1.5 h) and M_peak_←(6.9 ± 2.6 h) after shift schedule; Sleep onset did not significantly change in the free-run session		

C, control group; E, exercise group; LE, low-intensity exercise; HE, high-intensity exercise; CT0, time of awakening (e.g., CT3 indicates exercise starting 3 h after waking up); Wa, warm up; Ex, exercise; Re, rest; Co, cool down; VO_2peak_, maximum oxygen uptake; HR, heart rate; HRR, heart rate reserve; DLMO, dim light melatonin onset; M_onset_, Melatonin onset; M_peak_, Melatonin peak; M_offset_, Melatonin offset; aMT6s, 6- sulphatoxymelatonin (the major metabolite of melatonin); Arrows indicate significant phase advance (←) and delay (→).

### 2.1 Normal light/dark (LD) condition

Chronotypes refer to different phenotypes that are produced by individuals entraining different exogenous and endogenous factors (Fischer et al., 2017), which play a crucial role in health and shift work tolerance (Reiter et al., 2021). Based on these variations, people can be classified into morning types (early birds), evening types (night owls), and those with no extreme bias (Honkalampi et al., 2021). Studies have investigated the effects of morning and evening exercise on circadian rhythms in individuals with different chronotypes. Both morning and evening exercise advanced the sleep-wake cycle and dim light melatonin onset (DLMO) in night owls, while evening exercise delayed the phase of DLMO in early birds (Thomas et al., 2020). A questionnaire survey on a large sample size (N = 909) found that evening exercise led to a delayed sleep onset time than morning exercise, and the night owls were more significantly affected (Glavin et al., 2021).

The timing of exercise is a crucial factor to consider. For instance, under a condition of 16-h light:8-h dark (LD16:8), resistance exercise in the morning or noon can advance the circadian phase of core body temperature, whereas long-term rest in bed can delay it (Mendt et al., 2021). Furthermore, research on the relationship between children’s physical activity and sleep found that exercise time is related to sleep duration and efficiency (Antczak et al., 2021). Proper exercise during normal photoperiods can effectively regulate circadian rhythms, and it is recommended that the general population chooses morning exercise to improve sleep quality and advance sleep onset time.

### 2.2 Constant condition

In a constant environment without time cues, Miyazaki et al. found that 2 hours of moderate-intensity intermittent exercise (using a bicycle ergometer) in the afternoon (CT8, the timepoint of wake-up was defined as CT0) or night (CT17) delayed the onset of melatonin, while the timing of melatonin peak remained unchanged in the morning exercise group (CT2), when subjects were exposed to a constant dim light (<10 lux, equivalent to the indoor light intensity at dusk) (Miyazaki et al., 2021). Similarly, moderate-intensity intermittent aerobic exercise using a bicycle ergometer in the afternoon (CT10) or night (CT16) also delayed the onset of melatonin. However, when the exercise was replaced with 1-h high-intensity aerobic exercise, the melatonin peak levels increased without any changes in phases (Buxton et al., 1997; Yamanaka et al., 2015). In the same study, the authors found that cycling for 2 h in the morning (CT3) delayed the phase of melatonin peak by 1 h (Yamanaka et al., 2015). Additionally, Buxton et al. found that high-intensity exercise in the afternoon (CT10.5) advanced the peak phase of melatonin, while exercise at CT1.5, CT5, and CT16.5 delayed the peak phase of melatonin (Buxton et al., 2003).

These findings suggest that the impact of exercise on the circadian rhythm is influenced not only by the timing of exercise but also by other factors, such as exercise duration, intensity, and volume. However, even when studies use the same exercise protocol with the same timing, type, and intensity, the results may not be entirely consistent, which could be attributed to variations in sleep-wake schedules, small sample sizes, or significant individual differences.

### 2.3 Disturbed light/dark condition

In daily life, shift work, smartphone overuse, and long-haul flights across time zones may cause acute circadian disruption, suppressed melatonin production, and sleep deprivation (Wei et al., 2020; Roenneberg, 2023). To mitigate circadian disruption, people often attempt to synchronize their activities with natural time, adjust their sleep patterns and diet, or take melatonin supplements (Pfeffer et al., 2018). In recent years, more and more attention has been paid to the regulation of exercise on circadian rhythms. However, the impact of a single bout of exercise on circadian rhythms is relatively minor and much weaker than the effects of bright light exposure. Studies have shown that a single bout of exercise does not significantly influence the plasma melatonin (Minors et al., 1991; Buxton et al., 2003). In contrast, regular exercise over a prolonged period has a considerable impact on circadian rhythms, including the expression of core clock genes (Okamoto et al., 2013).

Studies investigating the impact of exercise on circadian disruption have employed various forced phase-shifted sleep-wake schedules. Under a forced schedule of 23.6-h sleep-wake cycle under dim light (<10 lux), Miyazaki et al. found that a 2-h cycling exercise at 3 and 7 h after waking advanced melatonin onset, which facilitate adaptation to the schedule, while the inactive group showed delayed phase of melatonin (Miyazaki et al., 2021). A study with an 8-h advanced phase shift has shown that cycling at 3-h and 7-h after waking for 4 consecutive days delayed the onset of melatonin and minimum core body temperature, which is opposite to the direction of phase shift (Yamanaka et al., 2010). In contrast, a few hours of intermittent bicycle exercise from CT7 for 3 consecutive days under a 9-hour-delayed sleep-wake schedule caused a delay in the phase of the minimum core body temperature. In addition, no difference was found between evening and morning types in the exercise groups, while the phase delay of the minimum core temperature was larger in the evening type in the control group.

**FIGURE 2:**
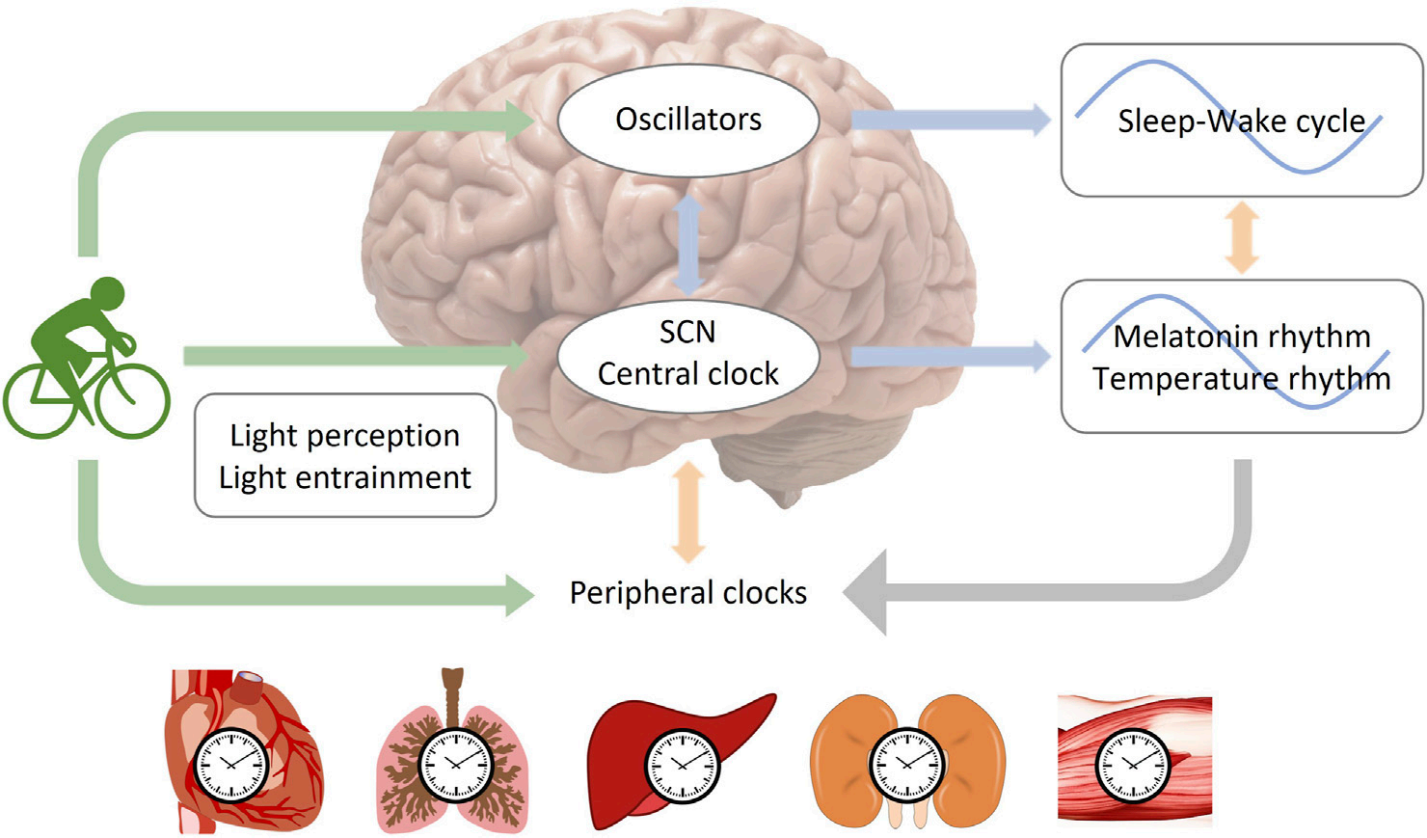
The entrainment of circadian rhythms by light and exercise. The central clock oscillator, which regulates rhythms of melatonin and body temperature, is located within the suprachiasmatic nucleus (SCN), while oscillators governing the sleep-wake cycle may exist in brain regions beyond the SCN (Yamanaka and Waterhouse, 2016; Yamanaka, 2020). Two potential pathways for entraining the central circadian rhythm through exercise are depicted: 1, exercise entrains the central clock directly; 2, exercise entrains oscillators in extra-SCN brain regions, which then transmit signals to the SCN. It is important to note that light serves as the most significant zeitgeber. The regulation of circadian rhythms by exercise can be influenced by the intensity of light, as exercise can impact the function of light perception or the entrainment process related to light.

Exercise during sleep time may negatively impact sleep quality due to increased body temperature and alertness (Baehr et al., 1999). Moderate or high-intensity aerobic cycling for 7 days was thought to synchronize melatonin rhythm with a 9-h delayed jet lag (Barger et al., 2004). Light intensity may also play a role in the effect of exercise on circadian rhythm. When subjects were exposed to bright light (>5,000 lux), cycling exercise was found to shift both melatonin and body temperature phases to the same direction as an 8-h advanced or 9-h delayed phase shift, while the control group showed desynchronization between melatonin and sleep-wake cycle (Baehr et al., 1999; Yamanaka et al., 2014). In the study simulating advanced jet lag, the time of exercise was set in the morning and the middle of the night, which may include both advance and delayed regions of the phase-response curves (PRC) (Yamanaka et al., 2014). As a result, the advanced shift of circadian rhythms in bright light may be due to exercise enhancing the entrainment of light to the circadian rhythm by regulating the 5-HT (5-hydroxytryptamine) system (Cymborowski, 1998; Mistlberger et al., 2010) or enhancing the light-sensing ability of the circadian system (Yamanaka and Waterhouse, 2016), which induced a larger advanced shift (Figure 2).

In another interesting study, participants were subjected to a 90-min ultrashort sleep (60 min)-wake (30 min) cycle in a laboratory setting, with low light intensity during wakefulness (50 lux) and sleep (0.5 lux). Under such a condition, each individual conducted a 60-min moderate treadmill per day during one of eight periods of wakefulness (at 3-h intervals from 01:00) for three consecutive days. The PRC of aMT6s (6- sulphatoxymelatonin) onset to exercise was plotted (Youngstedt et al., 2019). It was found that aMT6 onset was advanced by exercise at 07:00, 13:00, and 16:00 and delayed by exercise at 19:00 and 22:00. Such pattern was similar to a PRC of bright light (Kripke et al., 2007). A study using hamster housed under DD showed that a 2-h wheel running during CT4-CT11 advanced the phase, while exercise during CT23^−ΔΔCT^3 and CT17^−ΔΔCT^20 delayed it (Reebs and Mrosovsky, 1989). Comparison of the results between humans and hamsters showed that exercise during the active period often led to opposite phase shifts, possibly due to differences in habitual activity timing (Challet, 2007). Although the study on ultrashort sleep-wake cycles cannot fully predict the circadian rhythm phase shifts after exercise during normal sleep-wake schedules, it provides guidance for shift workers, who may need to avoid afternoon exercise and strong light exposure.

Regular exercise has been shown to promote synchronization between the sleep-wake cycle and the circadian clock, regardless of the direction of phase shift. The closer the exercise time was to the previous melatonin onset, the larger the phase shift in post-exercise melatonin onset (Barger et al., 2004; Yamanaka et al., 2010). However, previous studies have been limited by the protocols of schedules and exercise, making it difficult to compare results directly and identify the optimal timing of exercise for preventing or treating circadian disorders. Furthermore, the phenomenon of internal desynchronization suggests that the circadian pacemakers regulating melatonin and body temperature may be independent of the sleep-wake cycle, and that other brain regions outside of the SCN may be involved in regulating the sleep-wake cycle (Yamanaka, 2020). Further research is needed to investigate the interaction between different circadian pacemakers and whether exercise directly regulates the rhythm of the SCN, or indirectly by influencing the sleep-wake cycle.

**FIGURE 3:**
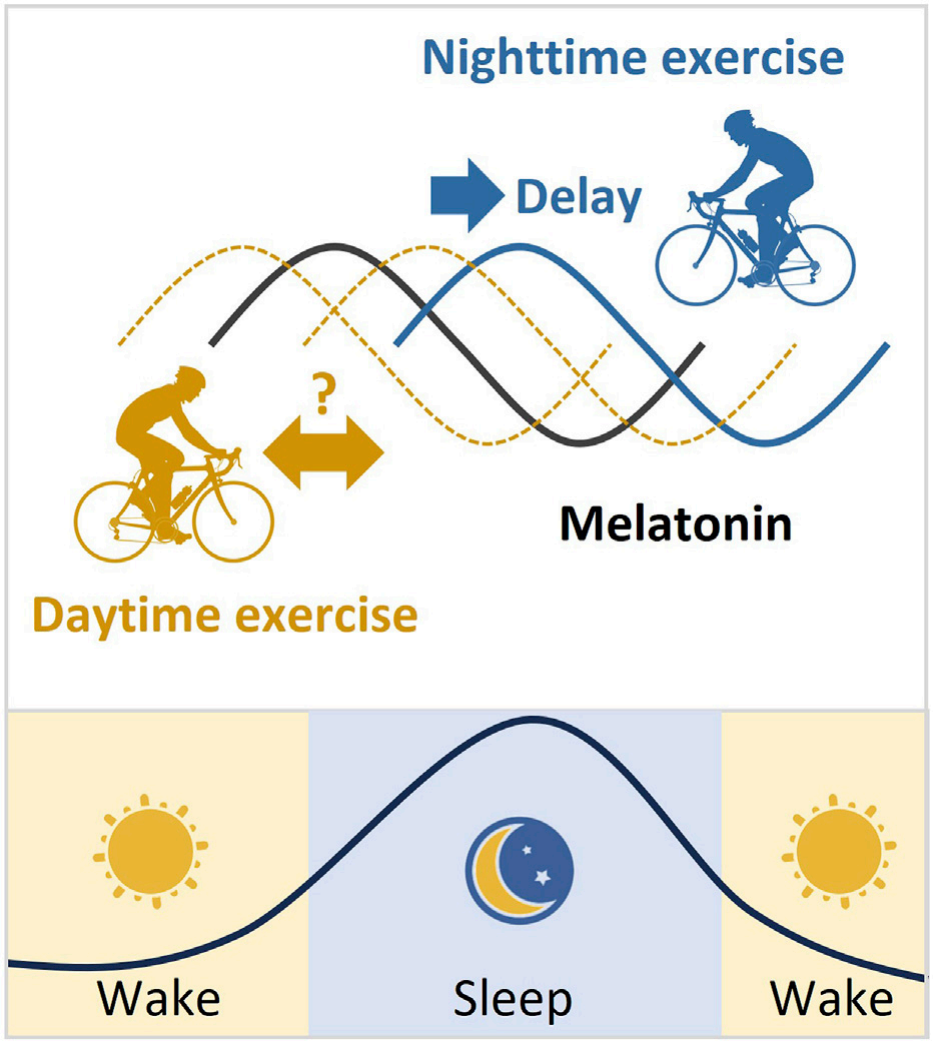
Melatonin rhythm and the phase changes after exercise. Melatonin is produced by the pineal gland. The onset of melatonin typically occurs 2–3 h before sleep onset and peaks in the middle of the night. Exercise at night usually delays the melatonin phase. However, the effect of daytime exercise on the melatonin rhythm is controversial.

In general, exercise plays an important role in regulating circadian rhythms. Exercise at night usually delays the circadian phase, which means it can make it harder to fall asleep at night and wake up in the morning. However, the effect of daytime exercise on circadian rhythms is more controversial (Figure 3). It is important to note that the effects of exercise on circadian rhythms can vary depending on several factors such as exercise intensity, mode, duration, energy supply, and frequency. Therefore, it is essential to consider these variables when designing an exercise regimen that aims to regulate the body’s internal clock.

Due to variations in detection methods and data processing, the results from studies of sleep-related circadian rhythm markers may be inconsistent. Activity accelerometers are often used for real-time detection of sleep, but only provide a rough estimate of sleep cycles as misjudgment of low-frequency activities may overvalue sleep time. While questionnaires are more convenient and suitable for large sample size research, they are relatively subjective. Additionally, the reliability of questionnaires is lower than that of activity accelerometers when investigating short-term sleep quality due to social factors influencing activity on work-free days. The most common marker of circadian rhythm detection in body fluids is DLMO, which can be detected in blood, saliva, and urine (Reiter et al., 2021). The criteria for determining the onset of DLMO can be divided into absolute and relative thresholds (Crowley et al., 2016). Different test samples and calculation methods used to evaluate the phase, amplitude, and period of circadian rhythm may lead to inconsistent results. Therefore, it is difficult to compare the intervention results of exercise on circadian rhythm at the same time systematically.

## 3 The relationship between exercise timing and diseases related to circadian disorders

Desynchronization between internal circadian rhythms and the environment can lead to various diseases. Disturbances in circadian rhythm not only affect sleep quality but also have a significant impact on energy metabolism, skeletal muscle, and vascular function in the body. Exercise can directly regulate disease-related physiological factors, or indirectly affect disease development by regulating circadian rhythm.

### 3.1 Sleep disorders

Sleep is mainly regulated by circadian rhythms and sleep-wake homeostasis. Sleep-wake homeostasis is determined as the driving force of sleep regulation, where the longer a person has been awake, the stronger the urge to sleep becomes. When the sleep pressure surpasses a threshold, it triggers sleep onset (Goel et al., 2013). The circadian wake-promoting signal interacts with the sleep-wake homeostasis to generate the sleep-wake cycle (Meyer et al., 2022). Primate experiments have shown that the circadian system plays a stronger role in regulating arousal than sleep-wake homeostasis and SCN lesions lead to an increase in total sleep time (Edgar et al., 1993), while exercise can interfere with sleep and wake behavior by influencing the input and output of SCN circadian signals and the expression of clock genes (Atkinson et al., 2007; Yamanaka, 2020).

It is well known that aerobic exercise, such as running for 30 min every morning for 3 weeks, is beneficial to sleep quality, mood, and concentration (Kalak et al., 2012). High-intensity interval exercise was shown to improve sleep quality as well (Min et al., 2021). Under disrupted light/dark schedules, certain exercise could also improve sleep and facilitate adaptation (Nakao et al., 2002; Yamanaka et al., 2010). In rodent models, increased brain-derived neurotrophic factor (BDNF) is associated with increased slow wave activity during sleep, and its related pathway is activated by endurance exercise (Faraguna et al., 2008; Wrann et al., 2013). As a nonphotic zeitgeber, regular exercise entrains the circadian rhythms at the molecular and physiological levels. On the other hand, sleep quality and the sleep-wake cycle can also affect exercise performance.

### 3.2 Cardiovascular diseases

Cardiovascular diseases (CVD) are the leading causes of death worldwide (WHO, 2021). Disruption of circadian rhythms increase the risk of cardiovascular diseases by increasing blood pressure and platelet aggregation (Chellappa et al., 2019). In a study involving CVD and type 2 diabetes patients who underwent 6 months of exercise, the expression of ALAS1, a CCG, increased in parallel with the diseases’ rehabilitation. (Steidle-Kloc et al., 2016). The variations in the *CLOCK* and *BMAL1* genes have been associated with an increased risk of cardiovascular diseases (CVD) (Corella et al., 2016; Škrlec et al., 2020). Additionally, BMAL1 plays a crucial role in vascular protection and angiogenesis, and its expression restored through exercise in aged endothelial cells (Sun et al., 2023).

Furthermore, timed exercise can appropriately reset the circadian system after circadian disruption to preserve cardiovascular health and exercise at evening is good for lowering blood pressure and heart rate (Brito et al., 2022). For instance, a 45-min aerobic exercise session in the evening, as opposed to the morning, led to a reduction in blood pressure by decreasing vasomotor sympathetic modulation and systemic vascular resistance in hypertensive individuals (Brito et al., 2019). In the case of patients with coronary artery disease, a 12-week regimen of evening walking produced more favorable outcomes than morning walking, resulting in lower levels of low-density lipoprotein cholesterol, fibrinogen, and white blood cell count (Lian et al., 2014). The risk of all-cause and CVD mortality was significantly reduced in the midday-afternoon and mixed moderate-to-vigorous intensity physical activity (MVPA) timing groups (Feng et al., 2023). Although one study linked morning exercise to a lower risk of CVD and stroke (Albalak et al., 2023), the mid-afternoon exercise subgroup was not included in this study. Overall, midday -afternoon and evening exercise is more commonly recognized as beneficial to cardiovascular health.

### 3.3 Cancer

The International Agency on Cancer (IARC) published an assessment in 2019 that classified shift work involving circadian disruption as “possibly carcinogenic to humans” (Ward et al., 2019). It is well known that long-term circadian disorders can affect the expression and activity of tumor suppressors and oncogenes, which disrupt homeostasis and increase the likelihood of tumors (Lee, 2021). The transcription, stability, and activity of p53, one of the most important tumor suppressor proteins, are regulated by BMAL1 and PER2 (Gotoh et al., 2014; Jiang et al., 2016). Additionally, overexpression of Per1 has been demonstrated to block the cell cycle in human cancer cells (Gery et al., 2006). Exercise has been found to have a positive impact on cancer prevention and treatment. For example, one study found that people who exercised in the morning had a lower risk of prostate and breast cancer (Weitzer et al., 2021). Patients with rectal cancer who engage in exercise during and after neoadjuvant chemoradiation demonstrate an elevated rate of pathologic complete response (Morielli et al., 2021). Exercise inhibited the growth of tumors and improved anti-cancer treatment efficacy. The pathway that prevents metastasis can be elicited through exercise-induced increase in cell damage, intratumoral metabolic stress, tumor perfusion and oxygen delivery (Hojman et al., 2018; Zhu et al., 2022). In addition, daily exercise at a fixed time was more beneficial for improving fatigue and quality of life in cancer survivors than irregular exercise (Coletta et al., 2021). The expression of core clock genes is regulated by exercise, but further research is needed to determine whether the effect of core clock genes on tumors is mainly attributed to their function of regulating circadian rhythm. Taking exercise as a non-drug approach to cancer will be an important direction in the development of sports medicine. It is necessary to explore exercise programs to maximize the prevention and treatment of cancer and its complications.

### 3.4 Metabolic diseases

In recent years, metabolic diseases such as diabetes and cardio- and cerebrovascular diseases are the leading causes of death worldwide (Fatima and Rana, 2020). Circadian disruption is a risk factor for metabolic syndrome (Chaput et al., 2023). Exercise has been shown to improve glucose and lipid metabolism, increase insulin sensitivity, and prevent or even reverse hyperglycemia, hyperlipidemia and hypercholesterolemia, which can reduce the risk of metabolic diseases and related complications partly by regulating circadian rhythms (Gabriel and Zierath, 2019; Murphy et al., 2020). A study found that 6-week moderate-intensity cycling before breakfast was three times more beneficial for lipid utilization than exercise after breakfast, and had greater benefits for improved insulin sensitivity and blood glucose (Edinburgh et al., 2020). This may be related to lower insulin levels during fasting exercise and the body’s tendency to use fat for the need of energy. Moreover, exercise can lead to higher irisin levels, which can promote the browning of adipocytes. This process increases the interaction between fat and muscle tissue, which can lead to improved metabolic function (Anastasilakis et al., 2014). Extensive changes in the expression of core clock genes including *Bmal1*, *Clock*, *Cry1/2*, *Per1/2/3*, *Rev-Erbα*, and *Rorα* have been observed after acute endurance exercise in mice (Maier et al., 2022). After early daytime exercise, but not early nighttime exercise, circadian associated repressor of transcription (*Ciart*) and *Per1* transcript were induced and involved in the regulation of skeletal muscle and liver metabolism (Maier et al., 2022). Transcriptome and metabolome analysis in mice showed that exercise during the early active period resulted in immediate changes to carbohydrate and adipose tissue metabolism, increased expression of genes associated with angiogenesis and glycolysis, and increased expression of genes associated with fatty acid oxidation, branched amino acid catabolism, and ketone metabolism (Sato et al., 2019; Pendergrast et al., 2023).

The above evidence suggests that exercise during the early active period may be more effective in treating metabolic disorders in both mice and humans. Nevertheless, it is important to acknowledge that several studies have indicated potential advantages of afternoon exercise in individuals with metabolic challenges. For example, in individuals with type 1 diabetes, post-exercise blood glucose levels were notably lower following afternoon resistance exercise (RE) compared to fasting morning RE (Toghi-Eshghi and Yardley, 2019). In men with type 2 diabetes, a 2-week regimen of afternoon high-intensity interval training (HIIT) not only led to more substantial improvements in blood glucose levels than morning HIIT but also resulted in an increase in thyroid-stimulating hormone (TSH) levels, accompanied by enhancements in mitochondrial content and skeletal muscle lipid profiles (Savikj et al., 2018; Savikj et al., 2022). Additionally, in overweight/obese men, evening exercise was associated with improved glycemic control and a partial reversal of metabolic changes induced by a high-fat diet (HFD), whereas morning exercise did not yield the same outcomes (Moholdt et al., 2021). These conflicting results indicate that the relationship between the timing of exercise and its effects on metabolism is complex and may vary depending on the exercise protocols, the population being studied and the specific metabolic outcome being measured. To gain a more comprehensive understanding of the relationship between exercise timing and metabolism, additional studies with more diverse experimental populations and time nodes are required.

### 3.5 Other diseases

Circadian disruption has been linked to diseases of all systems in the body, such as the musculoskeletal system, nervous system, and digestive system. Skeletal muscle is highly susceptible to aging, which leads to a loss of both mass and strength over time in elderly people (Wohlwend et al., 2021). This condition, known as sarcopenia, often results in falls, fractures, physical disabilities, and other harmful consequences (Cruz-Jentoft et al., 2019; Damluji et al., 2023). Besides aging, several other factors, including inadequate nutrition, inflammation, and disrupted circadian rhythms, may contribute to sarcopenia (Silva et al., 2021). Inflammation and circadian rhythms are well known to interact with each other, with proinflammatory factors impairing the function of clock genes that regulate muscle function and phenotype (Yang et al., 2013; Curtis et al., 2015; Schroder et al., 2015). Specifically, TNF-α upregulates the expression of core clock genes Bmal1 and Rorα while decreasing Rev-erbα (Yoshida et al., 2018), leading to disrupted circadian rhythms in skeletal muscle. Disruption of circadian rhythms can also intensify the oxidative stress and damage of neurons by compromising the neuroprotective effect of melatonin. Long-term circadian disruption can lead to cognitive impairment or dementia, and increase the risk of neurodegenerative diseases such as Alzheimer’s disease (Wu et al., 2019). It can also cause inflammation in the digestive system, leading to inflammatory bowel disease (Gombert et al., 2019).

As a non-photic factor regulating circadian rhythm, exercise can affect human health by regulating skeletal muscle and cardiopulmonary functions. Disruption of circadian rhythms in skeletal muscles is associated with an increased risk of chronic diseases, and the regulation of circadian rhythm-involved diseases by exercise is partly achieved by the regulation of skeletal muscle (Martin and Esser, 2022; Morrison et al., 2022; Wang et al., 2022). Maintaining a normal circadian rhythm is important for promoting skeletal muscle regeneration and repair, which can help prevent or alleviate muscular atrophy (Zhang et al., 2020). Both acute and long-term exercise can regulate the expression of clock genes in skeletal muscle and may improve circadian rhythms. Even low-intensity aerobic exercise can entrain the circadian rhythm of skeletal muscle (Choi et al., 2020; Saner and Lee, 2020). Exercise can also improve vascular health, whose deficiency is a potential risk factor for sarcopenia, by increasing the wall shear stress of arteries, stimulating endothelial cells to release nitric oxide (NO), and promoting vasodilation to improve nutrient supply to skeletal muscle (Shen et al., 2020). In addition, the timing of exercise has an impact on physical performance, as peak performance of aerobic exercise has been reported to occur later in the day, which is partly contributed by the diurnal fluctuations in mitochondrial function (Choi et al., 2020). Engaging in combined strength and endurance training during the evening may lead to greater gains in muscle mass compared to morning sessions (Küüsmaa et al., 2016). This suggests that exercise can be an effective strategy for preventing and managing sarcopenia by improving skeletal muscle function partly via maintaining circadian rhythms. However, further research is needed to fully understand the molecular mechanisms underlying the relationship between circadian rhythms, inflammation, and skeletal muscle function, and to identify optimal exercise interventions for preventing or treating sarcopenia.

The time of day is an important factor in maximizing the health benefits of exercise for disease prevention and treatment (Guan and Lazar, 2021; Bennett and Sato, 2023; Schönke et al., 2023). In summary, regular physical exercise plays a crucial role in preventing loss of muscle mass and strength by improving the immune system and vascular endothelial function, as well as synchronizing circadian rhythms of blood vessels and muscles. Further research is needed to better understand the mechanisms underlying the relationship between circadian rhythms, exercise, and diseases.

## 4 Conclusion

Exercise has garnered increasing attention as a significant non-photic zeitgeber. Summarized findings from this review of human data suggest that regular exercise can regulate the expression of clock genes, synchronize the circadian rhythm, and improve sleep health, metabolic and immune functions, thereby preventing and treating various diseases related to circadian disorder. Exercise at night usually delays the circadian phase, and the effect of daytime exercise on circadian rhythms is controversial. Midday-afternoon physical activity is associated with a lower all-cause and cardiovascular disease mortality, while morning exercise is connected to a decreased risk of cancer and improved lipid metabolism. The mechanism by which exercise affects the circadian rhythm needs to be further studied. In conclusion, determining the best timing and intensity of exercise for different populations is crucial to maximize the health benefits. Exercise holds great promise as a non-pharmacological intervention for preventing and treating circadian rhythm disorders and related diseases.

## References

[B1] Acosta-Rodriguez V. Rijo-Ferreira F. Izumo M. Xu P. Wight-Carter M. Green C. B. (2022). Circadian alignment of early onset caloric restriction promotes longevity in male C57BL/6J mice. Science 376 (6598), 1192–1202. 10.1126/science.abk0297 35511946 PMC9262309

[B2] Albalak G. Stijntjes M. van Bodegom D. Jukema J. W. Atsma D. E. van Heemst D. (2023). Setting your clock: Associations between timing of objective physical activity and cardiovascular disease risk in the general population. Eur. J. Prev. Cardiol. 30 (3), 232–240. 10.1093/eurjpc/zwac239 36372091

[B3] Anastasilakis A. D. Polyzos S. A. Saridakis Z. G. Kynigopoulos G. Skouvaklidou E. C. Molyvas D. (2014). Circulating irisin in healthy, young individuals: Day-night rhythm, effects of food intake and exercise, and associations with gender, physical activity, diet, and body composition. J. Clin. Endocrinol. Metab. 99 (9), 3247–3255. 10.1210/jc.2014-1367 24915120

[B4] Antczak D. Sanders T. Del Pozo Cruz B. Parker P. Lonsdale C. (2021). Day-to-day and longer-term longitudinal associations between physical activity, sedentary behavior, and sleep in children. Sleep 44 (4), zsaa219. 10.1093/sleep/zsaa219 33103724

[B5] Atkinson G. Edwards B. Reilly T. Waterhouse J. (2007). Exercise as a synchroniser of human circadian rhythms: An update and discussion of the methodological problems. Eur. J. Appl. Physiol. 99 (4), 331–341. 10.1007/s00421-006-0361-z 17165050

[B6] Baehr E. K. Fogg L. F. Eastman C. I. (1999). Intermittent bright light and exercise to entrain human circadian rhythms to night work. Am. J. Physiol. 277 (6), R1598–R1604. 10.1152/ajpregu.1999.277.6.R1598 10600904

[B7] Barger L. K. Wright K. P. Jr. Hughes R. J. Czeisler C. A. (2004). Daily exercise facilitates phase delays of circadian melatonin rhythm in very dim light. Am. J. Physiol. Regul. Integr. Comp. Physiol. 286 (6), R1077–R1084. 10.1152/ajpregu.00397.2003 15031136

[B8] Bennett S. Sato S. (2023). Enhancing the metabolic benefits of exercise: Is timing the key? Front. Endocrinol. (Lausanne) 14, 987208. 10.3389/fendo.2023.987208 36875451 PMC9974656

[B9] Bolshette N. Ibrahim H. Reinke H. Asher G. (2023). Circadian regulation of liver function: From molecular mechanisms to disease pathophysiology. Nat. Rev. Gastroenterology Hepatology. 10.1038/s41575-023-00792-1 37291279

[B10] Brito L. C. Marin T. C. Azevedo L. Rosa-Silva J. M. Shea S. A. Thosar S. S. (2022). Chronobiology of exercise: Evaluating the best time to exercise for greater cardiovascular and metabolic benefits. Compr. Physiol. 12 (3), 3621–3639. 10.1002/cphy.c210036 35766829 PMC10214902

[B11] Brito L. C. Pecanha T. Fecchio R. Y. Rezende R. A. Sousa P. Da Silva-Júnior N. (2019). Morning versus evening aerobic training effects on blood pressure in treated hypertension. Med. Sci. Sports Exerc 51 (4), 653–662. 10.1249/MSS.0000000000001852 30489494

[B12] Bruggisser F. Knaier R. Roth R. Wang W. Qian J. Scheer F. (2023). Best time of day for strength and endurance training to improve health and performance? A systematic review with meta-analysis. Sports Med. Open 9 (1), 34. 10.1186/s40798-023-00577-5 37208462 PMC10198889

[B13] Bull F. C. Al-Ansari S. S. Biddle S. Borodulin K. Buman M. P. Cardon G. (2020). World Health Organization 2020 guidelines on physical activity and sedentary behaviour. Br. J. Sports Med. 54 (24), 1451–1462. 10.1136/bjsports-2020-102955 33239350 PMC7719906

[B14] Buxton O. M. Frank S. A. L’Hermite-Baleriaux M. Leproult R. Turek F. W. Van Cauter E. (1997). Roles of intensity and duration of nocturnal exercise in causing phase delays of human circadian rhythms. Am. J. Physiol. 273 (3), E536–E542. 10.1152/ajpendo.1997.273.3.E536 9316443

[B15] Buxton O. M. Lee C. W. L’Hermite-Baleriaux M. Turek F. W. Van Cauter E. (2003). Exercise elicits phase shifts and acute alterations of melatonin that vary with circadian phase. Am. J. Physiology-Regulatory Integr. Comp. Physiology 284 (3), R714–R724. 10.1152/ajpregu.00355.2002 12571075

[B16] Chaix A. Zarrinpar A. Miu P. Panda S. (2014). Time-restricted feeding is a preventative and therapeutic intervention against diverse nutritional challenges. Cell Metab. 20 (6), 991–1005. 10.1016/j.cmet.2014.11.001 25470547 PMC4255155

[B17] Challet E. (2007). Minireview: Entrainment of the suprachiasmatic clockwork in diurnal and nocturnal mammals. Endocrinology 148 (12), 5648–5655. 10.1210/en.2007-0804 17901231

[B18] Chaput J. P. McHill A. W. Cox R. C. Broussard J. L. Dutil C. da Costa B. G. G. (2023). The role of insufficient sleep and circadian misalignment in obesity. Nat. Rev. Endocrinol. 19 (2), 82–97. 10.1038/s41574-022-00747-7 36280789 PMC9590398

[B19] Chellappa S. L. Vujovic N. Williams J. S. Scheer F. (2019). Impact of circadian disruption on cardiovascular function and disease. Trends Endocrinol. Metab. 30 (10), 767–779. 10.1016/j.tem.2019.07.008 31427142 PMC6779516

[B20] Chen L. Yang G. (2014). PPARs integrate the mammalian clock and energy metabolism. PPAR Res. 2014, 653017. 10.1155/2014/653017 24693278 PMC3945976

[B21] Choi Y. Cho J. No M. H. Heo J. W. Cho E. J. Chang E. (2020). Re-setting the circadian clock using exercise against sarcopenia. Int. J. Mol. Sci. 21 (9), 3106. 10.3390/ijms21093106 32354038 PMC7247148

[B22] Coletta A. M. Playdon M. C. Baron K. G. Wei M. Kelley K. Vaklavas C. (2021). The association between time-of-day of habitual exercise training and changes in relevant cancer health outcomes among cancer survivors. PLoS One 16 (10), e0258135. 10.1371/journal.pone.0258135 34637457 PMC8509995

[B23] Corella D. Asensio E. M. Coltell O. Sorlí J. V. Estruch R. Martínez-González M. Á. (2016). CLOCK gene variation is associated with incidence of type-2 diabetes and cardiovascular diseases in type-2 diabetic subjects: Dietary modulation in the PREDIMED randomized trial. Cardiovasc. Diabetol. 15 (1), 4. 10.1186/s12933-015-0327-8 26739996 PMC4704407

[B24] Crowley S. J. Suh C. Molina T. A. Fogg L. F. Sharkey K. M. Carskadon M. A. (2016). Estimating the dim light melatonin onset of adolescents within a 6-h sampling window: The impact of sampling rate and threshold method. Sleep. Med. 20, 59–66. 10.1016/j.sleep.2015.11.019 27318227 PMC4913029

[B25] Cruz-Jentoft A. J. Bahat G. Bauer J. Boirie Y. Bruyere O. Cederholm T. (2019). Sarcopenia: Revised European consensus on definition and diagnosis. Age Ageing 48 (1), 16–31. 10.1093/ageing/afy169 30312372 PMC6322506

[B26] Curtis A. M. Fagundes C. T. Yang G. Palsson-McDermott E. M. Wochal P. McGettrick A. F. (2015). Circadian control of innate immunity in macrophages by miR-155 targeting Bmal1. Proc. Natl. Acad. Sci. U. S. A. 112 (23), 7231–7236. 10.1073/pnas.1501327112 25995365 PMC4466714

[B27] Cymborowski B. (1998). Serotonin modulates a photic response in circadian locomotor rhythmicity of adults of the blow fly, Calliphora vicina. Physiol. Entomol. 23 (1), 25–32. 10.1046/j.1365-3032.1998.2310025.x

[B28] Damluji A. A. Alfaraidhy M. AlHajri N. Rohant N. N. Kumar M. Al Malouf C. (2023). Sarcopenia and cardiovascular diseases. Circulation 147 (20), 1534–1553. 10.1161/CIRCULATIONAHA.123.064071 37186680 PMC10180053

[B29] Deota S. Lin T. Chaix A. Williams A. Le H. Calligaro H. (2023). Diurnal transcriptome landscape of a multi-tissue response to time-restricted feeding in mammals. Cell Metab. 35 (1), 150–165.e4. 10.1016/j.cmet.2022.12.006 36599299 PMC10026518

[B30] Edgar D. M. Dement W. C. Fuller C. A. (1993). Effect of SCN lesions on sleep in squirrel monkeys: Evidence for opponent processes in sleep-wake regulation. J. Neurosci. 13 (3), 1065–1079. 10.1523/JNEUROSCI.13-03-01065.1993 8441003 PMC6576589

[B31] Edinburgh R. M. Bradley H. E. Abdullah N. F. Robinson S. L. Chrzanowski-Smith O. J. Walhin J. P. (2020). Lipid metabolism links nutrient-exercise timing to insulin sensitivity in men classified as overweight or obese. J. Clin. Endocrinol. Metab. 105 (3), 660–676. 10.1210/clinem/dgz104 31628477 PMC7112968

[B32] Fagiani F. Di Marino D. Romagnoli A. Travelli C. Voltan D. Di Cesare Mannelli L. (2022). Molecular regulations of circadian rhythm and implications for physiology and diseases. Signal Transduct. Target Ther. 7 (1), 41. 10.1038/s41392-022-00899-y 35136018 PMC8825842

[B33] Faraguna U. Vyazovskiy V. V. Nelson A. B. Tononi G. Cirelli C. (2008). A causal role for brain-derived neurotrophic factor in the homeostatic regulation of sleep. J. Neurosci. 28 (15), 4088–4095. 10.1523/jneurosci.5510-07.2008 18400908 PMC2597531

[B34] Fatima N. Rana S. (2020). Metabolic implications of circadian disruption. Pflugers Arch. 472 (5), 513–526. 10.1007/s00424-020-02381-6 32363530

[B35] Feng H. Yang L. Liang Y. Y. Ai S. Liu Y. Liu Y. (2023). Associations of timing of physical activity with all-cause and cause-specific mortality in a prospective cohort study. Nat. Commun. 14 (1), 930. 10.1038/s41467-023-36546-5 36805455 PMC9938683

[B36] Fischer D. Lombardi D. A. Marucci-Wellman H. Roenneberg T. (2017). Chronotypes in the US - influence of age and sex. PLoS One 12 (6), e0178782. 10.1371/journal.pone.0178782 28636610 PMC5479630

[B37] Fisk A. S. Tam S. K. E. Brown L. A. Vyazovskiy V. V. Bannerman D. M. Peirson S. N. (2018). Light and cognition: Roles for circadian rhythms, sleep, and arousal. Front. Neurol. 9, 56. 10.3389/fneur.2018.00056 29479335 PMC5811463

[B38] Gabriel B. M. Zierath J. R. (2019). Circadian rhythms and exercise — Re-setting the clock in metabolic disease. Nat. Rev. Endocrinol. 15 (4), 197–206. 10.1038/s41574-018-0150-x 30655625

[B39] Gery S. Komatsu N. Baldjyan L. Yu A. Koo D. Koeffler H. P. (2006). The circadian gene Per1 plays an important role in cell growth and DNA damage control in human cancer cells. Mol. Cell 22 (3), 375–382. 10.1016/j.molcel.2006.03.038 16678109

[B40] Glavin E. E. Ceneus M. Chanowitz M. Kantilierakis J. Mendelow E. Mosquera J. (2021). Relationships between sleep, exercise timing, and chronotype in young adults. J. Health Psychol. 26 (13), 2636–2647. 10.1177/1359105320926530 32498631

[B41] Goel N. Basner M. Rao H. Dinges D. F. (2013). Circadian rhythms, sleep deprivation, and human performance. Prog. Mol. Biol. Transl. Sci. 119, 155–190. 10.1016/B978-0-12-396971-2.00007-5 23899598 PMC3963479

[B42] Gombert M. Carrasco-Luna J. Pin-Arboledas G. Codoner-Franch P. (2019). The connection of circadian rhythm to inflammatory bowel disease. Transl. Res. 206, 107–118. 10.1016/j.trsl.2018.12.001 30615844

[B43] Gotoh T. Vila-Caballer M. Santos C. S. Liu J. Yang J. Finkielstein C. V. (2014). The circadian factor Period 2 modulates p53 stability and transcriptional activity in unstressed cells. Mol. Biol. Cell 25 (19), 3081–3093. 10.1091/mbc.E14-05-0993 25103245 PMC4230596

[B44] Guan D. Lazar M. A. (2021). Interconnections between circadian clocks and metabolism. J. Clin. Invest. 131 (15), e148278. 10.1172/JCI148278 34338232 PMC8321578

[B45] Hatori M. Vollmers C. Zarrinpar A. DiTacchio L. Bushong E. A. Gill S. (2012). Time-restricted feeding without reducing caloric intake prevents metabolic diseases in mice fed a high-fat diet. Cell Metab. 15 (6), 848–860. 10.1016/j.cmet.2012.04.019 22608008 PMC3491655

[B46] Hojman P. Gehl J. Christensen J. F. Pedersen B. K. (2018). Molecular mechanisms linking exercise to cancer prevention and treatment. Cell Metab. 27 (1), 10–21. 10.1016/j.cmet.2017.09.015 29056514

[B47] Honkalampi K. Jarvelin-Pasanen S. Tarvainen M. P. Saaranen T. Vauhkonen A. Kupari S. (2021). Heart rate variability and chronotype - a systematic review. Chronobiol Int. 38 (12), 1786–1796. 10.1080/07420528.2021.1939363 34130562

[B48] Jiang W. Zhao S. Jiang X. Zhang E. Hu G. Hu B. (2016). The circadian clock gene Bmal1 acts as a potential anti-oncogene in pancreatic cancer by activating the p53 tumor suppressor pathway. Cancer Lett. 371 (2), 314–325. 10.1016/j.canlet.2015.12.002 26683776

[B49] Kalak N. Gerber M. Kirov R. Mikoteit T. Yordanova J. Puhse U. (2012). Daily morning running for 3 weeks improved sleep and psychological functioning in healthy adolescents compared with controls. J. Adolesc. Health 51 (6), 615–622. 10.1016/j.jadohealth.2012.02.020 23174473

[B50] Koike N. Yoo S. H. Huang H. C. Kumar V. Lee C. Kim T. K. (2012). Transcriptional architecture and chromatin landscape of the core circadian clock in mammals. Science 338 (6105), 349–354. 10.1126/science.1226339 22936566 PMC3694775

[B51] Kripke D. F. Elliott J. A. Youngstedt S. D. Rex K. M. (2007). Circadian phase response curves to light in older and young women and men. J. Circadian Rhythms 5, 4. 10.1186/1740-3391-5-4 17623102 PMC1988787

[B52] Küüsmaa M. Schumann M. Sedliak M. Kraemer W. J. Newton R. U. Malinen J.-P. (2016). Effects of morning versus evening combined strength and endurance training on physical performance, muscle hypertrophy, and serum hormone concentrations. muscle hypertrophy, serum hormone concentrations 41 (12), 1285–1294. 10.1139/apnm-2016-0271 27863207

[B53] Lane J. M. Qian J. Mignot E. Redline S. Scheer F. Saxena R. (2023). Genetics of circadian rhythms and sleep in human health and disease. Nat. Rev. Genet. 24 (1), 4–20. 10.1038/s41576-022-00519-z 36028773 PMC10947799

[B54] Lee Y. (2021). Roles of circadian clocks in cancer pathogenesis and treatment. Exp. Mol. Med. 53 (10), 1529–1538. 10.1038/s12276-021-00681-0 34615982 PMC8568965

[B55] Li M. D. (2022). Clock-modulated checkpoints in time-restricted eating. Trends Mol. Med. 28 (1), 25–35. 10.1016/j.molmed.2021.10.006 34801412

[B56] Lian X.-Q. Zhao D. Zhu M. Wang Z.-M. Gao W. Zhao H. (2014). The influence of regular walking at different times of day on blood lipids and inflammatory markers in sedentary patients with coronary artery disease. Prev. Med. 58, 64–69. 10.1016/j.ypmed.2013.10.020 24201089

[B57] Maier G. Delezie J. Westermark P. O. Santos G. Ritz D. Handschin C. (2022). Transcriptomic, proteomic and phosphoproteomic underpinnings of daily exercise performance and zeitgeber activity of training in mouse muscle. J. Physiol. 600 (4), 769–796. 10.1113/JP281535 34142717 PMC9290843

[B58] Martin R. A. Esser K. A. (2022). Time for exercise? Exercise and its influence on the skeletal muscle clock. J. Biol. Rhythms 37 (6), 579–592. 10.1177/07487304221122662 36129164 PMC9729417

[B59] Martin R. A. Viggars M. R. Esser K. A. (2023). Metabolism and exercise: The skeletal muscle clock takes centre stage. Nat. Rev. Endocrinol. 19 (5), 272–284. 10.1038/s41574-023-00805-8 36726017 PMC11783692

[B60] Mendt S. Gunga H. C. Felsenberg D. Belavy D. L. Steinach M. Stahn A. C. (2021). Regular exercise counteracts circadian shifts in core body temperature during long-duration bed rest. NPJ Microgravity 7 (1), 1. 10.1038/s41526-020-00129-1 33402671 PMC7785743

[B61] Meyer N. Harvey A. G. Lockley S. W. Dijk D. J. (2022). Circadian rhythms and disorders of the timing of sleep. Lancet 400 (10357), 1061–1078. 10.1016/S0140-6736(22)00877-7 36115370

[B62] Min L. Wang D. You Y. Fu Y. Ma X. (2021). Effects of high-intensity interval training on sleep: A systematic review and meta-analysis. Int. J. Environ. Res. Public Health 18 (20), 10973. 10.3390/ijerph182010973 34682718 PMC8535574

[B63] Minors D. S. Waterhouse J. M. Wirz-Justice A. (1991). A human phase-response curve to light. Neurosci. Lett. 133 (1), 36–40. 10.1016/0304-3940(91)90051-t 1791996

[B64] Mistlberger R. E. Antle M. C. Glass J. D. Miller J. D. (2010). Behavioral and serotonergic regulation of circadian rhythms. Biol. Rhythm Res. 31 (3), 240–283. 10.1076/0929-1016(200007)31:3;1-k;ft240

[B65] Miyazaki T. Hashimoto S. Masubuchi S. Sato H. Honma K.-I. (2021). Phase-advance shifts of human circadian pacemaker areaccelerated by daytime physical exercise. Am. J. Physiol. Regul. Integr. Comp. Physiol. 281, R197–R205. 10.1152/ajpregu.2001.281.1.R197 11404294

[B66] Moholdt T. Parr E. B. Devlin B. L. Debik J. Giskeodegard G. Hawley J. A. (2021). The effect of morning vs evening exercise training on glycaemic control and serum metabolites in overweight/obese men: A randomised trial. Diabetologia 64 (9), 2061–2076. 10.1007/s00125-021-05477-5 34009435 PMC8382617

[B67] Morielli A. R. Usmani N. Boulé N. G. Severin D. Tankel K. Joseph K. (2021). Feasibility, safety, and preliminary efficacy of exercise during and after neoadjuvant rectal cancer treatment: A phase II randomized controlled trial. Clin. Colorectal Cancer 20 (3), 216–226. 10.1016/j.clcc.2021.05.004 34158253

[B68] Morrison M. Halson S. L. Weakley J. Hawley J. A. (2022). Sleep, circadian biology and skeletal muscle interactions: Implications for metabolic health. Sleep. Med. Rev. 66, 101700. 10.1016/j.smrv.2022.101700 36272396

[B69] Murphy R. M. Watt M. J. Febbraio M. A. (2020). Metabolic communication during exercise. Nat. Metab. 2 (9), 805–816. 10.1038/s42255-020-0258-x 32747791

[B70] Nakao M. Yamamoto K. Honma K. Hashimoto S. Honma S. Katayama N. (2002). A phase dynamics model of human circadian rhythms. J. Biol. Rhythms 17 (5), 476–489. 10.1177/074873002237141 12375623

[B71] Okamoto A. Yamamoto T. Matsumura R. Node K. Akashi M. (2013). An out-of-lab trial: A case example for the effect of intensive exercise on rhythms of human clock gene expression. J. Circadian Rhythms 11 (1), 10. 10.1186/1740-3391-11-10 24004634 PMC3846773

[B72] Pendergrast L. A. Lundell L. S. Ehrlich A. M. Ashcroft S. P. Schonke M. Basse A. L. (2023). Time of day determines postexercise metabolism in mouse adipose tissue. Proc. Natl. Acad. Sci. U. S. A. 120 (8), e2218510120. 10.1073/pnas.2218510120 36780527 PMC9974500

[B73] Pfeffer M. Korf H. W. Wicht H. (2018). Synchronizing effects of melatonin on diurnal and circadian rhythms. Gen. Comp. Endocrinol. 258, 215–221. 10.1016/j.ygcen.2017.05.013 28533170

[B74] Reebs S. G. Mrosovsky N. (1989). Effects of induced wheel running on the circadian activity rhythms of Syrian hamsters: Entrainment and phase response curve. J. Biol. Rhythms 4 (1), 39–48. 10.1177/074873048900400103 2519579

[B75] Reiter A. M. Sargent C. Roach G. D. (2021). Concordance of chronotype categorisations based on dim light melatonin onset, the morningness-eveningness questionnaire, and the Munich chronotype questionnaire. Clocks Sleep. 3 (2), 342–350. 10.3390/clockssleep3020021 34204181 PMC8293076

[B76] Ren B. Huang Y. Zhang J. Li J. Liu Z. Guan Y. (2022). Impact of time-restricted feeding on adaptation to a 6-hour delay phase shift or a 12-hour phase shift in mice. Nutrients 14 (15), 3025. 10.3390/nu14153025 35893879 PMC9329972

[B77] Ren B. Ma C. Chen L. FitzGerald G. A. Yang G. (2021). Impact of time-restricted feeding to late night on adaptation to a 6 h phase advance of the light-dark cycle in mice. Front. Physiol. 12, 634187. 10.3389/fphys.2021.634187 33664675 PMC7920952

[B78] Reppert S. M. Weaver D. R. (2002). Coordination of circadian timing in mammals. Nature 418 (6901), 935–941. 10.1038/nature00965 12198538

[B79] Roenneberg T. (2023). How can social jetlag affect health? Nat. Rev. Endocrinol. 19 (7), 383–384. 10.1038/s41574-023-00851-2 37221400 PMC10204006

[B80] Ruan W. Yuan X. Eltzschig H. K. (2021). Circadian rhythm as a therapeutic target. Nat. Rev. Drug Discov. 20 (4), 287–307. 10.1038/s41573-020-00109-w 33589815 PMC8525418

[B81] Saner N. J. Lee M. J. (2020). Exercise: it is only a matter of time. J. Physiol. 598 (21), 4755–4757. 10.1113/JP280366 32706397 PMC7689918

[B82] Sato R. Y. Yamanaka Y. (2023). Nonphotic entrainment of central and peripheral circadian clocks in mice by scheduled voluntary exercise under constant darkness. Am. J. Physiol. Regul. Integr. Comp. Physiol. 324, R526–R535. 10.1152/ajpregu.00320.2022 36802951

[B83] Sato S. Basse A. L. Schonke M. Chen S. Samad M. Altintas A. (2019). Time of exercise specifies the impact on muscle metabolic pathways and systemic energy homeostasis. Cell Metab. 30 (1), 92–110. 10.1016/j.cmet.2019.03.013 31006592

[B84] Savikj M. Gabriel B. M. Alm P. S. Smith J. Caidahl K. Björnholm M. (2018). Afternoon exercise is more efficacious than morning exercise at improving blood glucose levels in individuals with type 2 diabetes: A randomised crossover trial. Diabetologia 62 (2), 233–237. 10.1007/s00125-018-4767-z 30426166 PMC6323076

[B85] Savikj M. Stocks B. Sato S. Caidahl K. Krook A. Deshmukh A. S. (2022). Exercise timing influences multi-tissue metabolome and skeletal muscle proteome profiles in type 2 diabetic patients – a randomized crossover trial. Metabolism 135, 155268. 10.1016/j.metabol.2022.155268 35908579

[B86] Schloss M. J. Swirski F. K. Nahrendorf M. (2020). Modifiable cardiovascular risk, hematopoiesis, and innate immunity. Circ. Res. 126 (9), 1242–1259. 10.1161/CIRCRESAHA.120.315936 32324501 PMC7185037

[B87] Schönke M. Esser K. A. Gabriel B. M. (2023). Editorial: Circadian rhythms and exercise in cardiometabolic health. Front. Endocrinol. 14, 1180851. 10.3389/fendo.2023.1180851 PMC10070961 37025402

[B88] Schroder E. A. Harfmann B. D. Zhang X. Srikuea R. England J. H. Hodge B. A. (2015). Intrinsic muscle clock is necessary for musculoskeletal health. J. Physiol. 593 (24), 5387–5404. 10.1113/JP271436 26486627 PMC4704520

[B89] Shen B. Y. Liu H. B. Cao L. Qin K. R. (2020). Acute effects of different intensities of cycling acute exercise on carotid arterial apparent elasticity and hemodynamic variables. Biomed. Res. Int. 2020, 9027560. 10.1155/2020/9027560 33224984 PMC7669336

[B90] Silva B. S. A. Uzeloto J. S. Lira F. S. Pereira T. Coelho E. S. M. J. Caseiro A. (2021). Exercise as a peripheral circadian clock resynchronizer in vascular and skeletal muscle aging. Int. J. Environ. Res. Public Health 18 (24), 12949. 10.3390/ijerph182412949 34948558 PMC8702158

[B91] Škrlec I. Milić J. Steiner R. (2020). The impact of the circadian genes CLOCK and ARNTL on myocardial infarction. J. Clin. Med. 9 (2), 484. 10.3390/jcm9020484 32050674 PMC7074039

[B92] Steidle-Kloc E. Schönfelder M. Müller E. Sixt S. Schuler G. Patsch W. (2016). Does exercise training impact clock genes in patients with coronary artery disease and type 2 diabetes mellitus? Eur. J. Prev. Cardiol. 23 (13), 1375–1382. 10.1177/2047487316639682 27000098

[B93] Sun S. Ma S. Cai Y. Wang S. Ren J. Yang Y. (2023). A single-cell transcriptomic atlas of exercise-induced anti-inflammatory and geroprotective effects across the body. Innovation 4 (1), 100380. 10.1016/j.xinn.2023.100380 36747595 PMC9898793

[B94] Thomas J. M. Kern P. A. Bush H. M. McQuerry K. J. Black W. S. Clasey J. L. (2020). Circadian rhythm phase shifts caused by timed exercise vary with chronotype. JCI Insight 5 (3), e134270. 10.1172/jci.insight.134270 31895695 PMC7098792

[B95] Toghi-Eshghi S. R. Yardley J. E. (2019). Morning (fasting) vs afternoon resistance exercise in individuals with type 1 diabetes: A randomized crossover study. J. Clin. Endocrinol. Metabolism 104 (11), 5217–5224. 10.1210/jc.2018-02384 31211392

[B96] Ulgherait M. Midoun A. M. Park S. J. Gatto J. A. Tener S. J. Siewert J. (2021). Circadian autophagy drives iTRF-mediated longevity. Nature 598 (7880), 353–358. 10.1038/s41586-021-03934-0 34588695 PMC9395244

[B97] Valenzuela P. L. Castillo-Garcia A. Morales J. S. de la Villa P. Hampel H. Emanuele E. (2020). Exercise benefits on Alzheimer’s disease: State-of-the-science. Ageing Res. Rev. 62, 101108. 10.1016/j.arr.2020.101108 32561386

[B98] Wang J. Shen L. Zhang Y. Shen B. (2022). “Circadian rhythm and personalized exercise,” in Translational informatics: Sports and exercise medicine. Editor Shen B. (Singapore: Springer Nature Singapore), 99–122.

[B99] Ward E. M. Germolec D. Kogevinas M. McCormick D. Vermeulen R. Anisimov V. N. (2019). Carcinogenicity of night shift work. Lancet Oncol. 20 (8), 1058–1059. 10.1016/s1470-2045(19)30455-3 31281097

[B100] Wei T. Li C. Heng Y. Gao X. Zhang G. Wang H. (2020). Association between night-shift work and level of melatonin: Systematic review and meta-analysis. Sleep. Med. 75, 502–509. 10.1016/j.sleep.2020.09.018 33022488

[B101] Weitzer J. Castano-Vinyals G. Aragones N. Gomez-Acebo I. Guevara M. Amiano P. (2021). Effect of time of day of recreational and household physical activity on prostate and breast cancer risk (MCC-Spain study). Int. J. Cancer 148 (6), 1360–1371. 10.1002/ijc.33310 32976649 PMC7891656

[B102] WHO (2021). Cardiovascular diseases (CVDs). [Online] Accessed. Available: https://www.who.int/news-room/fact-sheets/detail/cardiovascular-diseases-(cvds).

[B103] Wohlwend M. Laurila P. P. Williams K. Romani M. Lima T. Pattawaran P. (2021). The exercise-induced long noncoding RNA CYTOR promotes fast-twitch myogenesis in aging. Sci. Transl. Med. 13 (623), eabc7367. 10.1126/scitranslmed.abc7367 34878822

[B104] Wolff C. A. Esser K. A. (2019). Exercise timing and circadian rhythms. Curr. Opin. Physiol. 10, 64–69. 10.1016/j.cophys.2019.04.020 31938759 PMC6959205

[B105] Wrann C. D. White J. P. Salogiannnis J. Laznik-Bogoslavski D. Wu J. Ma D. (2013). Exercise induces hippocampal BDNF through a PGC-1α/FNDC5 pathway. Cell Metab. 18 (5), 649–659. 10.1016/j.cmet.2013.09.008 24120943 PMC3980968

[B106] Wu H. Dunnett S. Ho Y. S. Chang R. C. (2019). The role of sleep deprivation and circadian rhythm disruption as risk factors of Alzheimer’s disease. Front. Neuroendocrinol. 54, 100764. 10.1016/j.yfrne.2019.100764 31102663

[B107] Xin H. Huang R. Zhou M. Chen J. Zhang J. Zhou T. (2023). Daytime-restricted feeding enhances running endurance without prior exercise in mice. Nat. Metab. 5, 1236–1251. 10.1038/s42255-023-00826-7 37365376

[B108] Yamanaka Y. (2020). Basic concepts and unique features of human circadian rhythms: Implications for human health. Nutr. Rev. 78 (12), 91–96. 10.1093/nutrit/nuaa072 33259616

[B109] Yamanaka Y. Hashimoto S. Masubuchi S. Natsubori A. Nishide S. Y. Honma S. (2014). Differential regulation of circadian melatonin rhythm and sleep-wake cycle by bright lights and nonphotic time cues in humans. Am. J. Physiol. Regul. Integr. Comp. Physiol. 307 (5), R546–R557. 10.1152/ajpregu.00087.2014 24944250

[B110] Yamanaka Y. Hashimoto S. Takasu N. N. Tanahashi Y. Nishide S. Y. Honma S. (2015). Morning and evening physical exercise differentially regulate the autonomic nervous system during nocturnal sleep in humans. Am. J. Physiol. Regul. Integr. Comp. Physiol. 309 (9), R1112–R1121. 10.1152/ajpregu.00127.2015 26333783

[B111] Yamanaka Y. Hashimoto S. Tanahashi Y. Nishide S. Y. Honma S. Honma K. (2010). Physical exercise accelerates reentrainment of human sleep-wake cycle but not of plasma melatonin rhythm to 8-h phase-advanced sleep schedule. Am. J. Physiol. Regul. Integr. Comp. Physiol. 298 (3), R681–R691. 10.1152/ajpregu.00345.2009 20042689

[B112] Yamanaka Y. Waterhouse J. (2016). Phase-adjustment of human circadian rhythms by light and physical exercise. J. Phys. Fit. Sports Med. 5 (4), 287–299. 10.7600/jpfsm.5.287

[B113] Yang G. Paschos G. Curtis A. M. Musiek E. S. McLoughlin S. C. Fitzgerald G. A. (2013). Knitting up the raveled sleave of care. Sci. Transl. Med. 5 (212), 212rv3. 10.1126/scitranslmed.3007225 24259052

[B114] Yoshida K. Nakai A. Kaneshiro K. Hashimoto N. Suzuki K. Uchida K. (2018). TNF-alpha induces expression of the circadian clock gene Bmal1 via dual calcium-dependent pathways in rheumatoid synovial cells. Biochem. Biophys. Res. Commun. 495 (2), 1675–1680. 10.1016/j.bbrc.2017.12.015 29217191

[B115] Youngstedt S. D. Elliott J. A. Kripke D. F. (2019). Human circadian phase-response curves for exercise. J. Physiol. 597 (8), 2253–2268. 10.1113/JP276943 30784068 PMC6462487

[B116] Zambon A. C. McDearmon E. L. Salomonis N. Vranizan K. M. Johansen K. L. Adey D. (2003). Time- and exercise-dependent gene regulation in human skeletal muscle. Genome Biol. 4 (10), R61. 10.1186/gb-2003-4-10-r61 14519196 PMC328450

[B117] Zhang H. Liang J. Chen N. (2020). Do not neglect the role of circadian rhythm in muscle atrophy. Ageing Res. Rev. 63, 101155. 10.1016/j.arr.2020.101155 32882420

[B118] Zhu C. Ma H. He A. Li Y. He C. Xia Y. (2022). Exercise in cancer prevention and anticancer therapy: Efficacy, molecular mechanisms and clinical information. Cancer Lett. 544, 215814. 10.1016/j.canlet.2022.215814 35803475

